# Association of Plasma Lipid Patterns and LDL Cholesterol Levels with Breslow Thickness and Ulceration in Melanoma Patients

**DOI:** 10.3390/ijms26041716

**Published:** 2025-02-17

**Authors:** István Szász, Viktória Koroknai, Tünde Várvölgyi, László Pál, Sándor Szűcs, Péter Pikó, Gabriella Emri, Eszter Janka, Imre Lőrinc Szabó, Róza Ádány, Margit Balázs

**Affiliations:** 1 HUN-REN-UD Public Health Research Group, Department of Public Health and Epidemiology, Faculty of Medicine, University of Debrecen, 4028 Debrecen, Hungary; szasz.istvan@med.unideb.hu (I.S.); adany.roza@med.unideb.hu (R.Á.); 2 Department of Public Health and Epidemiology, Faculty of Medicine, University of Debrecen, 4028 Debrecen, Hungary; koroknai.viktoria@med.unideb.hu (V.K.); pal.laszlo@med.unideb.hu (L.P.); szucs.sandor@med.unideb.hu (S.S.); piko.peter@med.unideb.hu (P.P.); 3 Department of Dermatology, Faculty of Medicine, University of Debrecen, 4032 Debrecen, Hungary; varvolgyi.tunde@med.unideb.hu (T.V.); gemri@med.unideb.hu (G.E.); janka.eszter@med.unideb.hu (E.J.); szabo.imre.lorinc@med.unideb.hu (I.L.S.)

**Keywords:** melanoma, Lipidyzer platform, plasma lipid profile, ulceration, Breslow depth, LDL-C, biomarkers

## Abstract

Recent evidence has highlighted the critical role of lipids in tumor biology. In this study, we analyzed the plasma lipid profiles of 151 melanoma patients (University of Debrecen, Department of Dermatology, Hungary) to examine the associations between specific lipid species and commonly used LDL-C lipid parameters, as well as the Breslow thickness and ulceration of primary tumors. Our analysis included patients who underwent primary tumor resection, comprising 83 individuals without metastases and 68 with metastases at the time of blood sampling. Lipid profiling was performed using the Lipidyzer™ platform, which targets over 1100 lipid species. Following quality control filtering, 802 lipids were included in the subsequent analyses. Ten lipids were found to have decreased plasma levels, while one lipid exhibited elevated plasma levels, both associated with an increased risk of higher Breslow thickness. Additionally, patients with thicker tumors (≥2 mm) demonstrated significantly higher LDL-C levels after adjusting for age, sex, therapy, and tumor presence (*p* = 0.032). Using forward stepwise regression, we identified a combination of four lipids—(CE(20:5), LCER(24:1), PE(P18:1/18:1), and LPE(18:2))—that demonstrated the strongest correlation with Breslow depth (AUC = 0.779, as determined by ROC analysis). Additionally, we identified 11 lipids significantly associated with tumor ulceration. Stepwise regression analysis further revealed two lipids (FFA(16:0) and PC(15:0/18:1)) capable of predicting tumor ulceration with an AUC score of 0.740. These findings suggest that individual lipid metabolism may influence tumor thickness and ulceration during the development and progression of primary melanoma.

## 1. Introduction

Early and accurate staging of malignant melanoma is essential for determining the prognosis of the disease and guiding the most appropriate treatment options. Among the various pathological parameters, including ulceration, mitotic rate, and lymph node involvement, Clark level and Breslow thickness have historically been significant in predicting outcomes of clinically localized primary malignant melanoma. Both were independently discovered in the late 1960s by Clark and Breslow [1,2,3,4]. The prognostic value of the Clark level is generally considered less significant compared to Breslow thickness [5], which has a strong correlation with patient survival and metastasis risk; therefore, it remains the primary prognostic indicator for the disease. Breslow depth is influenced by the combination of several factors including environmental (as varying sun exposure across different body parts), genetic factors, tumor localization, patient’s age and sex, and the presence of the tumor on the head and neck (likely due to the anatomical differences in the skin) [6,7,8]. Besides these factors, melanoma exhibits significant alterations in the overall metabolome, including lipid metabolism [9,10]. Metabolic reprogramming of cancer can influence tumor growth, progression, and response to treatment. The role of lipids, notably low-density lipoprotein (LDL), the primary carrier for cholesterol transportation in the blood, remains uncovered. In a recent article, Xie et al. aimed to describe novel insights into the role of LDL metabolism in melanoma progression and prognosis using single-cell sequencing, machine learning, and various other bioinformatics approaches [11]. Understanding the alterations of melanoma lipidome can help identify biomarkers and contribute to developing new targeted therapies, ultimately improving patient outcomes. In our recent study, we identified a panel of plasma lipid markers of patients with metastatic melanoma that are associated with the presence of metastasis, related to lymphogenous/hematogenous pathways, and correlated to patient survival [12]. In the present study, our aim was to discover the association between Breslow thickness and ulceration of primary melanoma patients’ lipidome using the same patient cohort and lipidome data obtained from the Lipidyzer™ quantitative lipidomics profiling platform, which allowed us to detect alterations in 13 lipid classes and more than 1100 lipid species. In addition, we investigated the correlation between Breslow thickness; treatment options; and the routinely measured blood biomarker, low-density lipoprotein-C (LDL-C).

Since none of our melanoma patients had a primary tumor at the time of blood sampling, we were able to detect lipid parameters significantly associated with primary tumor thickness and surface of the tumor, including routinely measured LDL-C, supporting our hypothesis that individual lipid metabolism affects the thickness and surface of the developing primary melanoma.

## 2. Results

### 2.1. Association Between LDL-C Level and Breslow Thickness of Melanoma Patients

**Figure 1:**
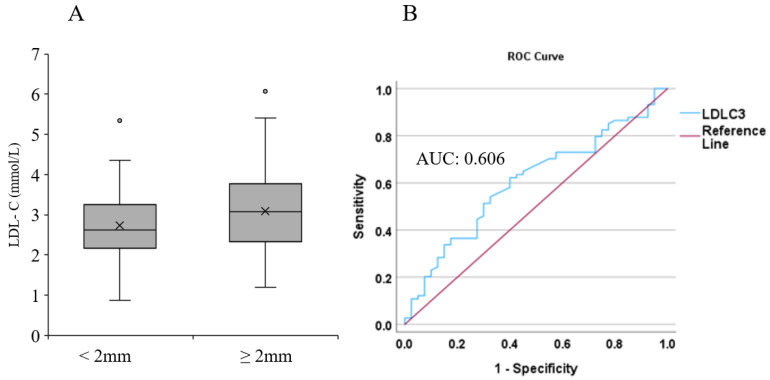
Association between LDL-C level and Breslow thickness of primary melanoma. (**A**) Association between LDL-C values and Breslow thickness of primary melanomas (<2 mm vs. ≥2 mm). (**B**) ROC curve analysis of LDL-C level and its association with Breslow thickness. The blue line represents LDL-C, and the red line indicates the reference line.

The LDL-C level was determined in 151 melanoma patients. The mean age at cohort entry was 62.9 (range, 29.9–84.3) years for men and 60.6 (range, 30.6–80.4) for women. The mean LDL-C levels were 2.78 nmol/L in men and 3.11 nmol/L in women. Melanoma patients were divided into two subgroups according to the Breslow thickness of the primary tumors (<2.00 mm and ≥2.00 mm). Compared to patients who had thinner primary melanoma (<2.00 mm; n = 41), those who had thicker primary melanoma (≥2.00 mm; n = 73) had higher LDL-C levels; however, this difference was not significant (*p* = 0.062; Figure 1A). Additional adjustment for age, gender, therapy, and the presence of tumor using a logistic regression model revealed that higher LDL-C value was significantly associated with the risk of a thicker primary tumor (OD: 1.786; CI: 1.087 to 2.935; *p* = 0.022). In the ROC curve, the area under the curve (AUC) for LDL-C level was 0.606 (Figure 1B).

### 2.2. Lipid Species Signature in Plasma Associated with Breslow Thickness of the Primary Tumor

**Figure 2:**
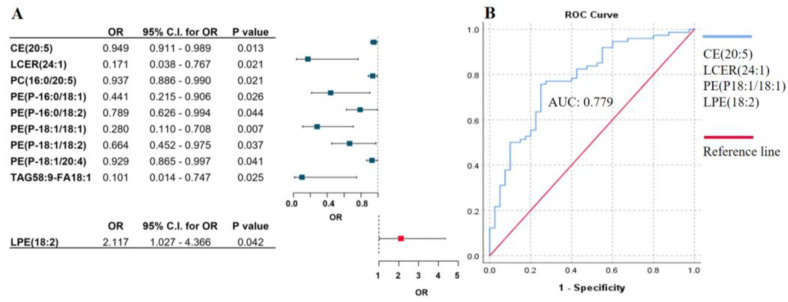
Association between lipid species pattern and Breslow thickness of primary melanoma. (**A**) Binary logistic regression analysis of lipid species in association with Breslow thickness of the primary tumors. Odds ratios and confidence intervals are visualized on a forest plot. Lipids with blue squares indicate a negative association, while lipids with red squares show a positive association with Breslow thickness of primary melanoma. Adjusted by sex, age, therapy, and the presence of tumor. (**B**) ROC curve analysis of the significant lipid panel identified by stepwise regression and its association with Breslow thickness. The blue line represents the lipid panel, and the red line indicates the reference line. OR: odds ratio, C.I.: confidence interval. Abbreviations: cholesteryl esters (CE), lactosylceramides (LCER), phosphatidylcholines (PC), phosphatidylethanolamines (PE), triacylglycerols (TAG), lysophosphatidylethanolamines (LPE).

In order to identify specific lipid patterns associated with the Breslow thickness, we analyzed plasma samples from 151 patients using the Lipidyzer platform, which targets more than 1100 lipid species. After quality control filtering, 802 lipids were included in the analysis. A logistic regression model was performed as described above; adjustment for age, gender, therapy, and the presence of tumor was added into the model. We found that nine lipids showed a negative association with Breslow thickness, indicating that patients with reduced levels of these lipids may have a higher risk for thicker primary tumors, and only one lipid (LPE(18:2)) exhibited an elevated plasma level in association with the risk of a higher Breslow value (Figure 2A). To select the lipids with the strongest predictive value, we applied forward stepwise regression. This analysis identified a combination of four lipids—(CE(20:5), LCER(24:1), PE(P18:1/18:1), and LPE(18:2))—with an AUC of 0.779 in ROC analysis (Figure 2B). The AUC value increased to 0.808 when the lipid panel was combined with LDL-C.

### 2.3. Association of Lipid Species Pattern with Ulceration of Primary Melanoma

**Figure 3:**
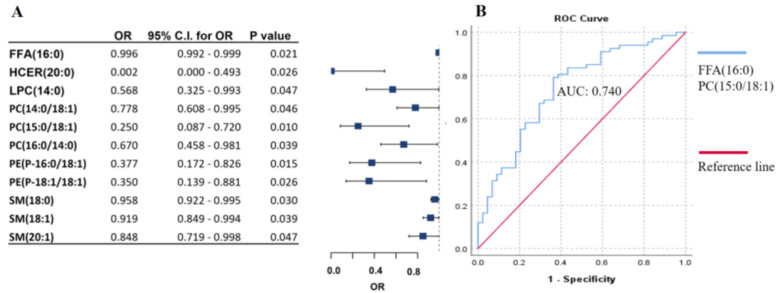
Association of lipid panels with ulceration of primary melanoma. (**A**) Binary logistic regression analysis of lipid species is negatively associated with ulceration of the primary tumor. The forest plot shows odds ratios and confidence intervals. (**B**) ROC curve analysis of the lipid panel associated with ulceration was identified by stepwise regression. The blue line represents the lipid panel created by stepwise regression, and the red line indicates the reference line. OR: odds ratio, C.I.: confidence interval. Adjusted by sex, age, therapy, and the presence of tumor. Free fatty acids (FFA), hexosylceramides (HCER), lysophosphatidylcholines (LPC), phosphatidylcholines (PC), phosphatidylethanol-amines (PE), sphingomyelins (SM).

Ulceration of the tumor tissue surface in melanoma patients refers to unfavorable clinical outcomes and ranks as the third most powerful predictor of survival, following tumor thickness and mitotic activity [13]. Patients samples were divided into two groups based on the presence or absence of ulceration of the primary tumor (ulcerated and non-ulcerated). Using the same method described above, we identified 11 lipids that were significantly associated with ulceration (Figure 3A). Among these lipids, stepwise regression analysis revealed two lipids, (FFA(16:0) and PC(15:0/18:1)), that can predict tumor ulceration with an AUC score of 0.740, as determined by ROC analysis (Figure 3B).

## 3. Discussion

It is widely accepted that the tumor microenvironment (TME) plays a major role in tumor development, progression, response to therapy, and drug resistance, and TME consists of various extracellular and cellular components [14]. In melanoma, perhaps in contrast to many tumors, the role of cancer-associated adipocytes (CAAs) is more pronounced, as they reach the subcutaneous fat layer during the invasion of the primary tumor [15]. During contact with tumor cells, adipocytes undergo both phenotypic and metabolomic changes. These cancer-associated adipocytes (CAAs) have tumor promoter effects and contribute to tumor growth, invasion, and metastasis formation through various factors, including cytokines, chemokines, adipokines, soluble lipid metabolites, and exosomes [16,17]. Furthermore, Coelho et al. published a very interesting article in which they described that the factors secreted by the subcutaneous and visceral fat layer differ greatly and, thus, have different effects on melanoma cells [18]. Not surprisingly, the subcutaneous adipocyte conditioned medium increased cell migration more, decreased cell adhesion, and increased colony forming ability compared to the visceral adipocyte conditioned medium, thereby better promoting tumor cell invasion [18].

In our study, we found nine lipid species whose reduced levels showed a significant correlation with the patient’s primary tumor thickness. Of these, six were phospholipids (five phosphatidylethanolamines (PEs) and one phosphatidylcholine (PC)), which are the primary building blocks of cell membranes [19]. PEs also function as lipid chaperone molecules and are essential for the initiation of apoptosis [20]. While phosphatidylethanolamines are found on the inner side of the normal cell and mitochondrial membrane, phospholipids and phosphatidylcholines are increased on the outer surface of tumors cells, making these lipids potential antitumor targets [21]. Due to increased cell division and metastasis formation, tumor cells have an increased need for these lipids to meet their increased cell membrane requirements. To do this, they either take up lipids from the circulation and/or synthesize lipids de novo, as shown by the overexpression of enzymes required for synthesis [9]. In our samples, the reduced concentration of PE and PC lipids in plasma may indicate that the tumors cells take up these lipids from circulation. Therefore, it is important to monitor changes in lipid metabolism at multiple levels (in tumor, in normal tissue, in circulation), as emphasized in our previous article [12,22]. Furthermore, we found a lipid with increased levels in association with thicker tumors: LPE(18:2). In the case of LPE(18:2), in their study of the platelet lipidome, Harm et al. found that elevated levels of this lipid were associated with an increased cardiovascular or bleeding risk [23]. It is possible that, in our case, these are also related to the fact that thicker tumors reach the blood vessels and thus possibly affect the lipidome of the platelets.

Ulceration of the tumor tissue surface in melanoma patients is linked to unfavorable clinical outcomes and is the third most powerful predictor of survival, following tumor thickness and mitotic activity [13]. Perhaps surprisingly, not all thick and proliferative tumors with high mitotic rates will have ulcerated surfaces. Therefore, we must consider other biological, molecular features of the tumors that may contribute to the loss of skin integrity [24]. It has also recently been shown that, according to the AJCC 8th Edition Melanoma Staging system, the two categories—“a” indicating no ulceration and “b” indicating ulceration—may not be entirely satisfactory. The extent and type of ulceration, as well as the involvement of the surrounding epidermis, provide more accurate prognostic information than merely its absence or presence [25,26]. Furthermore, ulceration is known to be more common in white males over 50 years of age, with risk factors of diabetes, smoking, low vitamin D levels, and higher BMI. The spindle phenotype of tumors cells is also significantly correlated with ulceration. At the molecular level, ulceration is associated with several proteins involved in epithelial–mesenchymal transition, antigen presentation, and autophagy (such as cadherin’s, PTEN, MHC1, and Beclin 1), as well as with changes in the expression of certain microRNAs. For example, microRNA-106-5b is increased in ulcerated melanoma, while microRNA-145-5p, microRNA-203-3p, microRNA-let-7b, and microRNA-1469 expression are decreased [13]. Despite these findings, a clear explanation for the development of ulceration remains elusive, and discovering any new markers in this field is crucial for the prognosis of the disease.

In our study, we identified lipids associated with ulceration that exhibited reduced plasma levels. Notably, among the 11 lipids identified, FFA(16:0), corresponding to palmitic acid, showed a significant decrease. Palmitic acid (FFA(16:0)) is one of the most common saturated fatty acids, derived from sources such as fish oils, milk fats, vegetable oils, and animal fats [27]. The lipid species HCER(20:0) has previously been linked to SARS-CoV-2 and HIV infections [28,29]; however, its association with tumor progression has not yet been reported. LPC(14:0) has been identified as a possible biomarker for drug-induced interstitial lung disease [30]. Additionally, the presence of ulceration correlated with reduced levels of three sphingomyelin species: (SM(18:0), SM(18:1), SM(20:1)). Łuczaj et al. found that sphingomyelin species tended to decrease in the plasma of rats irradiated with UVA and UVB, raising the possibility that the presence of ulceration in melanomas may also be related to UV exposure [31].

Finally, we found that LDL-C levels significantly correlated with the Breslow thickness of primary melanomas. Cholesterol levels varied among different tumor types. The lowest values were observed in patients with tumors in the hepatopancreaticobiliary system, while the highest values were found in melanoma, cerebral tumors, and breast cancers. Additionally, significantly lower cholesterol levels were noted in deceased patients. The lowest cholesterol levels were measured in tumors with the highest mitotic rates such as mesenchymal tumors, cerebral tumors, and breast cancer [32].

It is indeed true that, for breast tumors, it has been described that patients with high LDL-C levels are more prone to larger tumors, higher differentiation, and higher proliferation rates. However, to our knowledge, such an association has not been described for melanoma [33,34]. Our data are also consistent with the observation that the use of statins does not reduce the risk of developing melanoma but is associated with lower Breslow thickness [35]. These two observations are consistent with each other and support our hypothesis that individual lipid metabolism affects the thickness and surface of the developing primary melanoma.

## 4. Materials and Methods

### 4.1. Melanoma Patients and Tumor Samples

All tumor samples were processed in compliance with the guidelines and regulations of the University of Debrecen, Hungary, and with approval from the Ethics Committee of the Hungarian Scientific Council for Health (TUKEB 17876–2018/EKU and BMEÜ/715-1/2022/EKU). This study included the same cohort of 151 melanoma patients previously reported [12]; all samples were collected in the Department of Dermatology, Faculty of Medicine, University of Debrecen, Debrecen, Hungary. The characteristics of the tumor samples are summarized in Table 1.

Eighty-three patients were considered virtually metastasis-free, having had a negative CT scan at least one month prior to blood sampling, indicating the absence of metastasis. Additionally, 68 patients already had metastatic melanoma at the time of blood sampling. The patients involved 84 males (56%) and 67 (44%) females with a median age of 61.91 years (range 29–84 years). Based on the Breslow thickness, tumor samples were grouped into the following groups: <2 mm (n = 40) vs. ≥2 mm (n = 74); we did not have available data for 37 primary melanomas. Ulceration of the primary tumors was detected on 67 melanomas, and the surface of 44 tumors displayed no ulceration; in 40 samples, no data were available. The number of patients who received targeted therapy (TAFINLAR^®^+MEKINIST^®^ or Zelboraf+COTELLIC^®^) and immunotherapy (OPDIVO^®^+YERVOY^®^ or Keytruda) were the same, n = 87 each. Of the patients, 64 did not receive any therapy (Table 1).

### 4.2. Blood Samples and Extraction of Lipids from Plasma

Blood samples were obtained from the Department of Dermatology, Faculty of Medicine, University of Debrecen, Hungary. Specimens were transferred to the Department of Public Health and Epidemiology on dry ice and were stored at −80 °C until use. Collection and handling of blood samples, preparing the plasma, and lipid extraction were described in detail in our previous study [12]. All of the reagents were of HPLC grade. Internal standard (ISTD) kits (containing ISTDs for 13 lipid classes), pike standards with quality control plasma kits, SelexION tuning kits, and system suitability test kits for quantitative lipidomic analysis of human samples were purchased from AB Sciex Germany GmbH (Darmstadt, Germany). The composition of ISTD standard mixtures containing isotope-labeled lipid molecules was previously described in detail [36,37]. Lipids were extracted from the plasma samples using a modified Bligh–Dyer method [38].

### 4.3. Lipidomic Analysis and Data Processing

Analyses of lipid samples were conducted using HPLC coupled with electrospray ionization in tandem with mass spectrometry (HPLC ESI-MS-MS), as was described in detail previously [36]. The Lipidyzer platform consisting of a Nexera X2 HPLC (Shimadzu Germany GmbH, Duisburg, Germany) and a Sciex QTRAP 5500 system equipped with SelexION technology (AB Sciex Germany GmbH, Darmstadt, Germany) was used for lipidomic analysis. NanoViper capillary tubes with dimensions of 750 × 0.05 mm and 350 × 0.05 mm (Thermo Fisher Scientific Inc., Waltham, MA, USA) were used to connect the HPLC auto sampler valve to the grounding union and the grounding union to the ESI electrode (internal diameter: 65 µm).

To measure lipid concentrations, 50 µL of each sample was injected using flow injection at a rate of 7.0 µL/min. The running solution was a 1:1 mixture of dichloromethane and methanol containing 10 mM ammonium acetate. To minimize sample carryover, zero dead volume capillary tubes were used, and capillaries were cleaned with a dichloromethane–methanol mixture (1:1) at a flow rate of 30 µL/min for 2 min between injections. Each sample underwent two separate analyses—first with SelexION differential mobility spectrometric separation (DMSS) and then without it. The total run time for each sample was 21 min, including injector washing, sample injection, measurement in positive and negative ion modes, and post-run capillary washing. DMSS separates lipids based on their specific head group dipole moments, with varying compensation voltages (COVs) enabling the sequential analysis of different lipid classes. To optimize the separation process, 1-propanol was added as a chemical modifier to the curtain gas. The DMSS settings were as follows: low temperature, separation voltage of 3.5 kV, and low spectrometric resolution. Lipid species were detected and quantified using multiple reaction monitoring with automatic switching between positive and negative ion modes.

Negative ion mode with DMSS was used for analyzing PCs, PEs, and LPCs, while FAs were measured without DMSS. Positive ion mode with DMSS was used for SMs, whereas TGs, DGs, CEs, and Cers were analyzed without DMSS. The mass spectrometer was configured with the following settings: curtain gas set to 17, collision gas set to medium, ion spray voltage at 4.1 kV in positive mode and −2.5 kV in negative mode, temperature at 200 °C, nebulizer gas at 17, and heater gas at 25. Quality control (QC) and QC spike samples were included in each batch of eight plasma samples. System operation, data acquisition, and analysis were managed using Lipid Workflow Manager software (Software Version: 1.0.5.0(10/15/2018); A B Sciex Germany GmbH, Darmstadt, Germany), which automatically provided lipid concentrations in nmol/g of plasma. Weekly maintenance included manual cleaning of the differential mobility unit, orifice plate, and QJet Ion Guide using a 1:1 mixture of dichloromethane and methanol. After cleaning, the differential mobility unit was recalibrated with the SelexION tuning kit, followed by a system suitability test to ensure optimal performance.

The nomenclature of lipids proposed by the Lipid Maps Consortium was followed in this study [39].

### 4.4. Statistical Analysis

Statistical analyses followed the methods of our previous work [12]. The Shapiro–Wilk test was used to assess the normality of data. Multivariable logistic regression analysis was used to assess the associations between lipid levels and prognostic factors (e.g., presence of metastases, mortality, and location of metastases), adjusting for age, sex, and type of therapy (no therapy, immunotherapy, or targeted therapy). Odds ratios (ORs) with 95% confidence intervals (CIs) were calculated. Stepwise regression (forward method) was used to identify the main lipid species that were independently significantly associated with prognostic factors (after adjustment for sex, age, and therapeutic approaches). ROC curve and AUC (area under the curve) were calculated to assess the predictive performance of lipid species and their combination. Estimation of the combined AUC of lipid species with individually significant associations was assessed using the predicted probability value calculated by binary logistic regression analyses. Comparisons of lipid levels were performed using the Mann–Whitney U and Kruskal–Wallis tests, with Dunn’s test for post hoc analysis. Statistical analyses were carried out using IBM SPSS Statistics 26.0 software (IBM company, Palo Alto, CA, USA) or R 3.6.1 software (R Foundation for Statistical Computing, Vienna, Austria). *p* < 0.05 was considered statistically significant.

## 5. Conclusions

Altered lipid synthesis, uptake, and storage in melanoma cells provide energy and signaling molecules that promote tumor growth, invasion, and survival. The present study highlights significant alterations in lipid profiles associated with ulceration and Breslow thickness in malignant melanoma. Using the state-of-the-art quantitative lipidomics profiling platform, Lipidyzer™, we identified a panel of lipids with reduced plasma levels in ulcerated melanomas, including FFA(16:0), palmitic acid, and three sphingomyelin species—(SM(18:0), SM(18:1), and SM(20:1))—suggesting a possible link to UV exposure and tumor progression. The potential biomarker LPC(14:0) and the unexplored role of HCER(20:0) in cancer progression warrant further investigation. Additionally, we observed a significant correlation between LDL-C levels and Breslow thickness, indicating a complex role for lipid metabolism in melanoma growth. Our findings align with previous observations that statin use is associated with reduced Breslow thickness, supporting the hypothesis that individual lipid metabolism influences the growth and characteristics of primary melanomas. Our results provide new insights into the metabolic alterations in melanoma and suggest that lipid profiling could serve as a valuable tool for understanding tumor progression and identifying potential biomarkers for prognosis and therapeutic intervention.

## Figures and Tables

**Table 1:** Characteristics of melanoma patients and tumor samples.

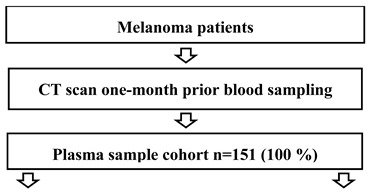			
Tumor Free Patients		Patients with Metastasis	
Number of Patients (%)			
	83 (54.97)		68 (45.03)
**Gender**		**Gender**	
Female	44 (29.14)	Female	23 (15.23)
Male	39 (25.83)	Male	45 (29.80)
**Age groups (average age: 61.10 years)**		**Age groups (average age: 63.04 years)**	
20–50	22 (14.57)	20–50	7 (4.64)
≥50	61 (40.40)	≥50	61 (40.40)
**Breslow thickness**		**Breslow thickness**	
<2 mm	30 (19.87)	<2 mm	10 (6.62)
≥2 mm	40 (26.49)	≥2 mm	34 (22.51)
No data	13 (8.61)	No data	24 (15.89)
**Ulceration**		**Ulceration**	
Present	32 (21.19)	Present	35 (23.18)
Absent	34 (22.52)	Absent	10 (6.62)
No data	17 (11.26)	No data	23 (15.23)
**Type of therapy at the time of blood sampling**			
None	53 (35.10)	None	11 (7.28)
Immunotherapy ^a^	22 (14.57)	Immunotherapy ^a^	42 (27.81)
Targeted therapy ^b^	8 (5.30)	Targeted therapy ^b^	15 (9.93)

^a^ Immunotherapies: OPDIVO^®^ (nivolumab; Bristol-Myers Squibb Company, Princeton, NJ 08543 USA); OPDIVO^®^ (nivolumab)+YERVOY^®^ (ipilimumab; Bristol-Myers Squibb Company, USA); KEYTRUDA (pembrolizumab; Merck Sharp & Dohme LLC Rahway, NJ 07065, USA). ^b^ Targeted therapies: TAFINLAR^®^ (dabrafenib; Novartis Pharmaceuticals Corporation East Hanover, NJ 07936, USA)+MEKINIST^®^ (trametinib; Novartis Pharmaceuticals, USA); ZELBORAF (vemurafenib; Genentech, San Francisco, CA, USA, Inc., A Member of the Roche Group, 1 DNA Way, South San Francisco, CA 94080-4990, USA)+COTELLIC^®^ (cobimetinib; Genentech USA, Inc, USA).

## Data Availability

The data presented in this study are available on request from the corresponding author. The data are not publicly available due to ethical restrictions.
